# Thick and Thin Filament Gene Mutations in Striated Muscle Diseases

**DOI:** 10.3390/ijms9071259

**Published:** 2008-07-16

**Authors:** Homa Tajsharghi

**Affiliations:** Department of Pathology, Sahlgrenska University Hospital, S-413 45 Göteborg, Sweden Tel.: +46-31-3422343; Fax: +46-31-417283; E-Mail: homa.tajsharghi@gu.se

**Keywords:** sarcomere, myosin, myosin heavy chain, actin, myopathy, myosin myopathy

## Abstract

The sarcomere is the fundamental unit of cardiac and skeletal muscle contraction. During the last ten years, there has been growing awareness of the etiology of skeletal and cardiac muscle diseases originating in the sarcomere, an important evolving field. Many sarcomeric diseases affect newborn children, i. e. are congenital myopathies. The discovery and characterization of several myopathies caused by mutations in myosin heavy chain genes, coding for the major component of skeletal muscle thick filaments, has led to the introduction of a new entity in the field of neuromuscular disorders: *myosin myopathies*. Recently, mutations in genes coding for skeletal muscle thin filaments, associated with various clinical features, have been identified. These mutations evoke distinct structural changes within the sarcomeric thin filament. Current knowledge regarding contractile protein dysfunction as it relates to disease pathogenesis has failed to decipher the mechanistic links between mutations identified in sarcomeric proteins and skeletal myopathies, which will no doubt require an integrated physiological approach. The discovery of additional genes associated with myopathies and the elucidation of the molecular mechanisms of pathogenesis will lead to improved and more accurate diagnosis, including prenatally, and to enhanced potential for prognosis, genetic counseling and developing possible treatments for these diseases. The goal of this review is to present recent progress in the identification of gene mutations from each of the major structural components of the sarcomere, the thick and thin filaments, related to skeletal muscle disease. The genetics and clinical manifestations of these disorders will be discussed.

## 1. Introduction

During the past decade, major advances have been made in defining the molecular basis of many genetically transmitted diseases. During this period, there has been growing awareness of the importance of sarcomeric protein mutations in the etiology of myopathy. Numerous mutations are currently associated with myopathy, with remarkable spectrum of phenotypic variation. In addition to a better understanding of the primary defect and basic molecular pathogenesis of disease, redefining the diagnostics of many disorders is another benefit of identifying the disease genes. These fundamental approaches will allow us to understand why a point mutation at one site in a sarcomeric protein (e.g. tropomyosin (TM)) leads to nemaline myopathy (NM), while a different point mutation causes distal arthrogryposis (DA). Improved understanding of the molecular basis of disease will probably allow targeting of pharmacological strategies as well as providing the cornerstone for gene therapy approaches.

In this review, disorders caused by mutation of sarcomeric thick and thin filament proteins will be discussed.

## 2. The Sarcomere

**Figure 1.**
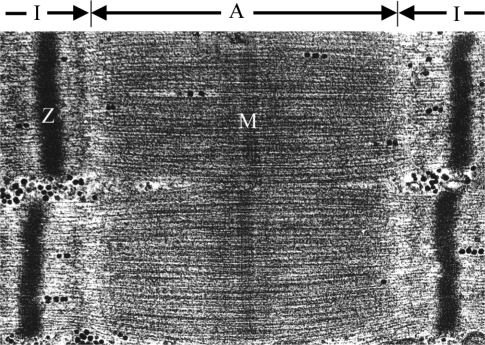
a) Ultrastructurally, the A-band corresponds to the thick filaments and includes a zone where the thin filaments overlap the thick filaments. The I-band is the zone in which the thin filaments do not overlap the thick filaments. The Z-line is a dark band in the centre of the I-band and the M-line runs down the center of the A-band.

The sarcomere represents the basic contractile unit of both skeletal and cardiac muscle. It is a highly ordered structure composed of the thin and thick filaments, titin, and nebuline [1]. The characteristic striated appearance of muscle fibers is observable by electron microscopy as alternating light (I) and dark (A) bands (Figure 1). The principle components of striated muscle sarcomeres include parallel arrays of actin-containing thin filaments that span the I-band and overlap with myosin-containing thick filaments in the A-band. The thin filaments are anchored in the Z-disc and the thick filaments are similarly anchored in the M-band [1].

**Figure 2.**
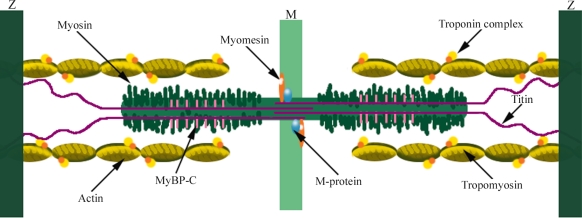
Illustration of the I-band, A-band, and M-line regions of the sarcomere. The thin filaments contain actin, tropomyosin, troponins C, I, and T and nebulin. The thick filaments are composed of myosin with the globular heads forming cross-bridges with thin filaments. Myosin-binding proteins, including MyBP-C, are associated with the thick filaments. The giant protein titin extend the length of an entire half sarcomere. The M-line contains different proteins, such as myomesin and M-protein.

Skeletal muscle function depends on a precise alignment of actin and myosin filaments. This is achieved by accessory proteins, such as α-actinin, myomesin, M-protein, titin, desmin and myosin-binding proteins (MyBP)-C and -H, which link the different components and keep them aligned with each other [1] (Figure 2). The major component of the Z-line is α-actinin, which acts as an actin cross-linking protein and holds actin filaments in a lattice arrangement in the Z-disc [2, 3]. It has been proposed that myomesin and M-protein may connect titin and myosin filament systems and that myomesin plays a role in integrating thick filaments into assembling sarcomeres [4]. Titin, a huge protein which runs parallel to the filament array, forms a continuous filament system in myofibrils [1]. Desmin is the predominant intermediate filament protein of striated muscle [5] and contributes to maintaining the integrity and alignment of myofibrils [1]. MyBP-C is localized in seven stripes running parallel to the M-band (Figure 2). Because MyBP-C interacts with both the thick and titin filaments, its function may be to link them together and/or to align the thick filaments in the A-band [1]. In addition, MyBP-C reduces the critical concentration for myosin polymerization and the resulting filaments are longer and more uniform in length than those polymerized in its absence [6, 7]. Both myosin-binding proteins appear to aid in the assembly of vertebrate muscle thick filaments into their precise lengths [1]. It has also been suggested that MyBP-C and –H may be involved in regulating muscle contraction [8].

### 2.1. The Major Component of the Thick Filament: Myosin

Contraction of muscle is the result of cyclic interactions between the globular heads of the myosin molecules, also known as cross-bridges, and the actin filaments. The repetitive binding and release results in sliding of the thick filaments along the thin filaments powered by the hydrolysis of ATP. Myosin can be regarded as an ATPase that is activated by the binding of actin [9].

**Figure 3.**
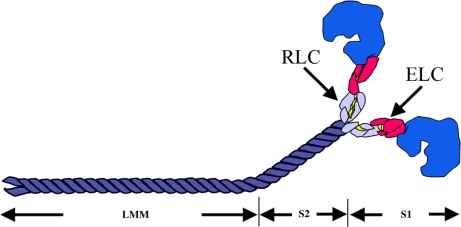
Schematic illustration of a myosin class II molecule, showing the essential light chains (ELC) and regulatory light chains (RLC) that wrap around the α-helical region of the S1. Two MyHC molecules intertwine via their α-helical regions to form a coiled-coil rod. Proteolytic fragments S1, S2 and LMM are indicated.

Myosin acts as a molecular motor that converts the chemical energy of ATP hydrolysis into mechanical force in eukaryotic cells [10]. Conventional myosin exists as a hexameric protein composed of two myosin heavy chain (MyHC) subunits and two pairs of non-identical light chain (MyLC) subunits (Figure 3). The MyHC has two functional domains. The globular, amino-terminal head domain, to which MyLCs bind, exhibits the motor function. The elongated alpha-helical coiled-coil carboxyl-terminal rod domain exhibits filament-forming properties [10]. The globular head that forms the cross-bridges contains the binding sites for actin and ATP [11].

The MyHC can be cleaved by proteolytic enzymes into two subfragments, heavy meromyosin (HMM) and light meromyosin (LMM) [12, 13]. The HMM contains the head region, termed subfragment 1 (S1), and a portion of the coiled-coil-forming sequence referred to as subfragment 2 (S2) which connects the myosin heads to the thick filament. The LMM is the C-terminal proportion of the rod, which lies along the thick filAMent axis [14] (Figure 3).

**Figure 4.**
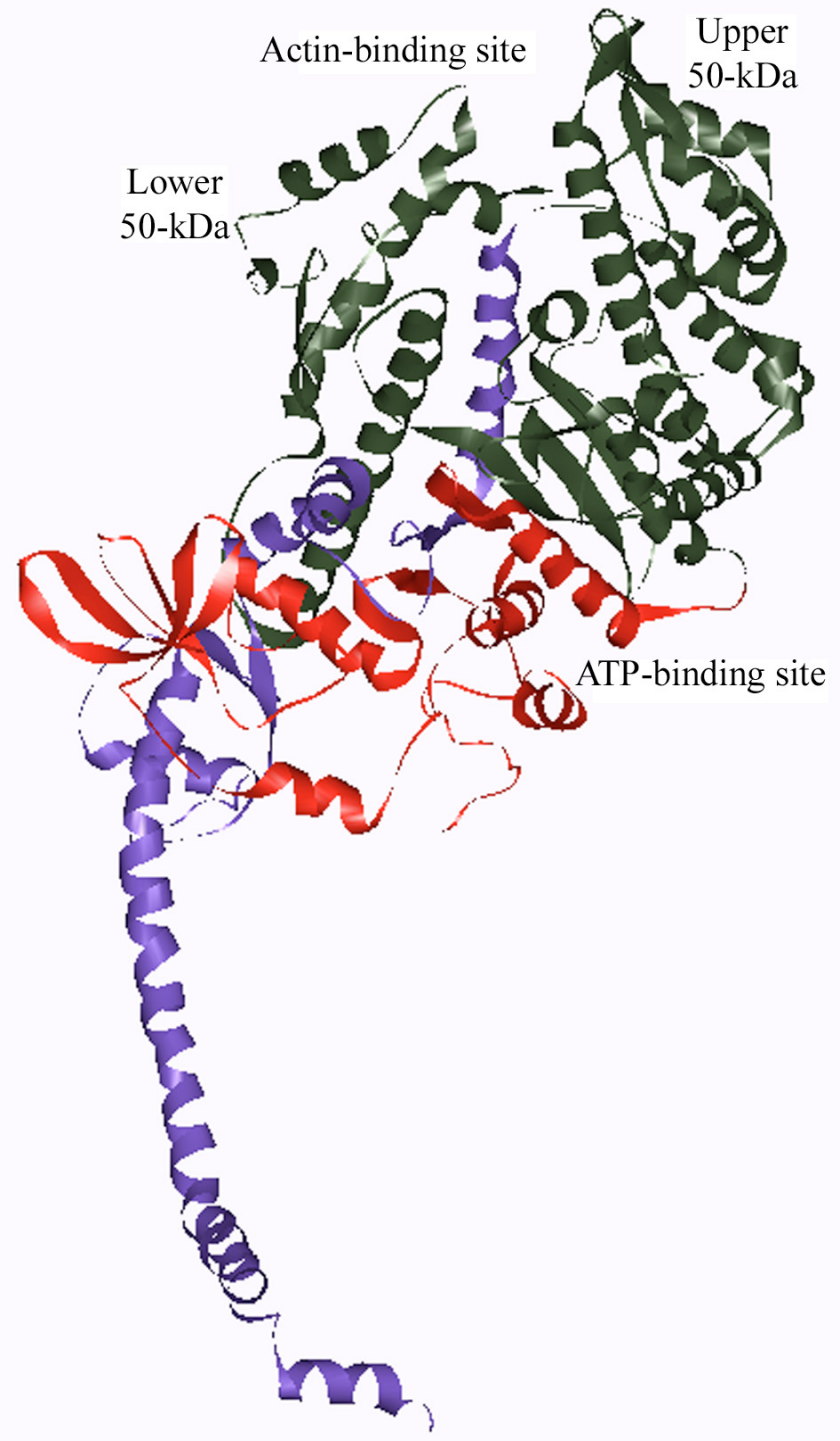
Ribbon representation of the chicken myosin S1 structure. The 25-, 50-, and 20-kDa segments of the heavy chain are shown as red, green, and purple ribbons, respectively. The upper and lower segments of the 50-kDa domain are indicated. The ATP- and actin-binding sites are shown. This figure was prepared using the program WebLab VIEWERLIFE 3.2.

The myosin motor domain is essentially constructed of three domains connected by flexible linkers. The 25-kDa amino-terminal nucleotide-binding domain is connected to the upper 50 kDa subdomain which is, in turn, connected to the lower 50-kDa subdomain. The third, 20-kDa domain is called the converter region (Figure 4). A long helix emerges from the converter domain and serves as the binding site for MyLCs. The essential light chains (ELC) occupy the binding site closest to the converter domain while the regulatory light chains (RLC) occupy the second site [15]. The binding sites are highly specific for their respective MyLCs [14]. The elongated neck region in S1 has been suggested to act as a lever arm to amplify small changes in the configuration of the motor domain into much larger displacements of actin [15].

Six striated muscle MyHCs are encoded by genes found in a tightly linked cluster on human chromosome 17 [16, 17]. The genes are arranged in the following order: *MYH3, MYH2, MYH1, MYH8, MYH13* and *MYH4* [18]. Cardiac MyHC isoforms encoded by *MYH7* and *MYH6* are located on chromosome 14 [19].

Three major MyHC isoforms are present in adult human limb muscle tissue: MyHC I, also called slow/beta MyHC (encoded by *MYH7*) is expressed in slow, type 1 muscle fibers and in heart ventricles; MyHC IIa (encoded by *MYH2)* is expressed in fast, type 2A muscle fibers and MyHC IIx (encoded by *MYH1*) is expressed in fast, type 2B muscle fibers [20]. In addition to the common MyHC isoforms found in fibers of adult human limb muscles, there are special MyHC isoforms expressed in some fibers in specific muscles. For example, very rapidly contracting fibers that express a specific MyHC isoform, extraocular MyHC, have been found in extraocular muscles [21–24]. Developing and regenerating muscle fibers express special MyHC isoforms, *i.e.* embryonic (encoded by *MYH3*) and perinatal (encoded by *MYH8*) MyHC. Embryonic MyHC is normally not expressed in postnatal human limb muscles unless there is ongoing muscle regeneration.

### 2.2. The Major Components of the Thin Filament: Actin, Tropmyosin, Troponin Complex and Nebulin

**Figure 5.**
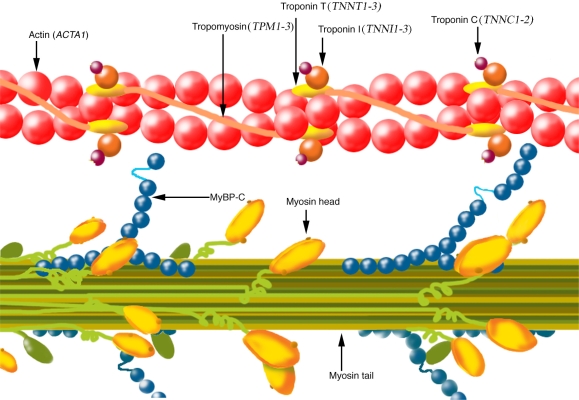
Schematic illustration of a part of the sarcomere, the contractile unit of muscle, composed of thick and thin filaments. Note that the illustration does not correspond to a notion of size.

The thin filament is the main site of Ca^+2^ regulation and is composed of four components in striated muscle: actin, TM and troponin (Tn) with its three subunits [25] (Figure 5).

Actin is the principal protein component of the sarcomeric thin filaments. F-actin forms the backbone of the thin filament which can be viewed as a two-stranded helical structure [25].

TM is an actin-binding protein, composed of two α-helical chains forming a rod-shaped coiled-coil dimer. TM is one of the regulatory proteins in the thin filament and is localized head-to-tail along the length of the actin filament, providing stability; it is essential for myosin–actin interaction [25, 26]. Through Ca^2+^-dependent movement transmitted via the Tn complex, TM blocks or opens the myosin binding sites on actin [25]. So far, four different TM genes have been identified in the human genome, *TPM1, TPM2, TPM3* and *TPM4* [26].

There are three highly homologous major TM isoforms in human striated muscle: α-TM(α–TM_fast_), β-TM and γ-TM (α–TM_slow_) [26].

α-TM is a product of the *TPM1* gene, β-TM is encoded by the *TPM2* gene and γ-TM is encoded by the *TPM3* gene. The muscle isoform encoded by *TPM1* is predominantly expressed in cardiac muscle and fast, type 2 muscle fibers. *TPM2* is mainly expressed in slow, type 1 and, to some extent, in fast muscle fibers and cardiac muscle. *TPM3* is predominantly expressed in slow muscle fibers and is also expressed in the heart [27].

Tn exists as a complex of three component proteins: troponin C (TnC), troponin I (TnI) and troponin T (TnT). Each of the three proteins is a regulator of muscle contraction and plays distinct roles in the thin filament. TnC is the Ca^2+^ -binding subunit, TnI is the inhibitory subunit that binds to actin and inhibits actomyosin ATPase, and TnT is the TM-binding subunit that links the Tn complex to TM [25].

There are two TnC isoforms in human striated muscle, expressed by two separate genes, *TNNC1* and *TNNC2* [28, 29]. The product of the *TNNC1* gene expressed in slow twitch skeletal muscle is also the isoform expressed in heart. The fast skeletal muscle TnC isoform is the product of *TNNC2*.

There are three TnI isoforms that are the products of separate genes in human striated muscle: fast skeletal TnI is the product of *TNNI2*, slow skeletal TnI is a product of *TNNI1* and cardiac TnI is a product of *TNNI3* [30].

There are three homologous TnT genes that encode specific TnT isoforms in humans: slow skeletal muscle TnT (encoded by *TNNT1*), fast skeletal muscle TnT (encoded by *TNNT3*) and cardiac TnT (encoded by *TNNT2*) [31, 32].

Nebulin is a giant filamentous protein extending along the entire length of the thin filament, recognized as a length-regulating template of the sarcomeric actin filaments, with single nebulin polypeptides spanning the length of the actin filament [33–35].

## 3. Myopathies Involving Thick and Thin Filament Proteins

During the last decade, several protein components of the thick and thin filaments have been implicated in the pathogenesis of muscle diseases: slow/β-cardiac myosin heavy chain (MyHC I), fast type IIa myosin heavy chain (MyHC IIa), embryonic myosin heavy chain (MyHC-emb), perinatal myosin heavy chain (MyHC-peri), skeletal muscle alpha actin (α-actin), α-tropomyosin slow (γ-TM), β-tropomyosin (β-TM), slow TnT, fast TnT and fast TnI.

Furthermore, there are additional myopathies associated with mutations in the genes coding for other proteins of the sarcomere such as titin, desmin and components in the Z-disk, which is not included in this review.

### 3.1. Disorders-associated Mutations Found in Thick Filament Components

#### 3.1.1. Myosin Heavy Chains

Mutation in the sarcomeric motor protein MyHC was first described in 1990 in association with a severe form of hypertrophic cardiomyopathy (HCM) [36].

During the past few years, MyHC mutations have been associated with different skeletal muscle diseases including autosomal dominant myopathy with congenital joint contractures, ophthalmoplegia and rimmed vacuoles (OMIM #605637), caused by a single point mutation in the fast IIa MyHC gene (*MYH2*) [37]; Laing early onset distal myopathy (OMIM #160500) [38–41] and myosin storage myopathy (OMIM #608358) [42–46], caused by different mutations in the slow/β-cardiac MyHC gene (*MYH7*); trismus-pseudocamptodatyly syndrome (TPS) (OMIM #158300), caused by mutation in perinatal MyHC (*MYH8*) [47, 48]; Freeman-Sheldon syndrome (FSS) (OMIM #193700) and Sheldon-Hall syndrome (SHS) (OMIM #601680), caused by mutations in embryonic MyHC (*MYH3*) [49].

##### 3.1.1.1. MyHC IIa

Mutation of a MyHC gene was not described in association with pure skeletal muscle until 2000 [37]. This myopathy, also called “Autosomal dominant MyHC IIa myopathy” (OMIM #605637), is associated with a missense mutation in the MyHC IIa gene (*MYH2*). Clinical characteristics are congenital joint contractures, which normalize during early childhood, external ophthalmoplegia and predominantly proximal muscle weakness and atrophy. The course is frequently progressive in adulthood [50]. The mutation changes a highly conserved, negatively charged glutamate at position 706 into a positively charged lysine (E706K). The altered amino acid is located in the SH1 helix in the core of the myosin head.

Three additional mutations in the *MYH2* gene have later been identified with myopathies in three families [51].

##### 3.1.1.2. Slow/β-cardiac MyHC

Mutations in the cardiac/β-MyHC gene (*MYH7*) are a frequent cause of familial hypertrophic/dilated cardiomyopathy (HCM/DCM) [52, 53]. More than 190 *MYH7* mutations with varying clinical penetrance have been described, some with relatively benign effects on life expectancy and others associated with a high incidence of sudden death (http://www.hgmd.cf.ac.uk/ac/gene.php?gene=MYH7). Despite the knowledge that the slow/β-cardiac MyHC gene is also expressed in slow, type 1 skeletal muscle fibers, only a few studies have investigated the involvement of skeletal muscle in familial hypertrophic cardiomyopathy patients with identified missense mutations in the *MYH7* gene [54, 55].

Laing early onset distal myopathy is identified and linked to the *MYH7* gene on chromosome 14q11 [38]. Of the total number of identified mutations associated with this form of distal myopathy, the most prevalent are mutations located in the LMM region of the myosin tail of slow/β-cardiac MyHC [38–41]; only two cases of distal myopathy with cardiomyopathy have been associated with a mutation in the S1 region [56, 57].

Myosin storage myopathy is an additional myopathy associated with mutations in the *MYH7* gene. It has been assigned various descriptive terms such as “myopathy with probable lysis of thick filaments” [58] and “hyaline body myopathy” [59, 60]. This myopathy is characterized by accumulation of slow/β cardiac myosin (MyHC I) in type I muscle fibers. Myosin storage myopathy has so far been associated with four mutations located in the distal rod region of slow/β-cardiac MyHC. The Arg1845Trp mutation has been reported in several unrelated cases [42, 44, 45]. This mutation has also been reported in cases with scapulo-peroneal myopathy [61].

##### 3.1.1.3. Perinatal MyHC

To date, only one single mutation in perinatal MyHC (*MYH8*), R674Q, has been associated with TPS (OMIM #158300). The mutation has been identified in seven unrelated families with this disorder [47, 48]. TPS is a rare autosomal dominant distal arthrogryposis (DA7), characterized by camptodactyly of the fingers, apparent only upon dorsiflexion of the wrist (pseudocamptodactyly), and inability to completely open the mouth (trismus) [62].

##### 3.1.1.4. Embryonic MyHC

DA syndromes are a group of sporadic and/or autosomal dominant disorders. The clinical features are multiple congenital contractures, where the hands and feet are tightly clenched and the fingers overlap [62, 63]. FSS, the most severe disorder with multiple congenital contractures and severe contractures of the orofacial muscles, and SHS, the most common DA, have recently been associated with mutations in the embryonic MyHC gene (*MYH3*) [49]. All of the *MYH3* mutations associated with FSS except one are located in the myosin head. Interestingly, 6/20 investigated cases in a study of FSS carried an R672 substitution, which is paralogous to the R674 of perinatal MyHC (*MYH8*) [49]. However, the mutations that cause SHS are located throughout *MYH3* [49].

A new study has recently reported novel *MYH3* mutations associated with DA and demonstrated myopathic changes in muscle biopsy specimens from patients with DA and *MYH3* mutations [64]. The results of this study add *MYH3* to the list of MyHC genes involved in hereditary myosin myopathies.

#### 3.1.2. Myosin Light Chains

##### 3.1.2.1. Regulatory Light Chain RLC

Recent studies have shown that myosin RLC is one of the sarcomeric proteins associated with FHC [65–69]. Some of the RLC mutations were shown to be associated with a particular type of FHC, [67] whereas others presented with a more classic form of FHC, resulting in increased left ventricular wall thickness and abnormal ECG [65, 66, 68, 69].

Since the RLC gene (*MYL2*), like *MYH7*, is expressed in slow skeletal muscle, skeletal muscle biopsies from patients with the Glu22Lys *MYL2* mutation exhibited abnormal skeletal muscle histology, similar to patients with ragged red fiber (RRF) myopathy [67].

##### 3.1.2.2. Essential Light Chain ELC

Mutations in the ELC have been associated with FHC. The pathological phenotypes vary in severity but almost all ELC mutations result in sudden cardiac death at a young age [70].

The *MYL3* gene encodes the slow skeletal and the ventricular ELC (ELC_v_) isoforms, whereas the *MYL4* gene encodes atrial ELC (ELC_a_) [71]. Morphological analyses of skeletal muscle biopsies from patients with FHC associated with the Met149Val *MYL3* mutation also revealed a similar RRF histology [67].

### 3.2. Disorder-associated Mutations Found in Thin Filament Components

#### 3.2.1. Actin

Mutations in the skeletal muscle alpha actin gene (*ACTA1*) have been associated with various skeletal muscle diseases, including actin myopathy (accumulation of actin), NM, intranuclear rod myopathy and congenital fiber type disproportion (CFTD) [72–74]. To date, more than 80 different *ACTA1* mutations have been identified, distributed throughout the entire alpha actin gene. Most of these mutations are associated with NM. NM is defined by the presence of rod-shaped structures in muscle fibers, so-called nemaline rods, largely composed of α-actinin and actin. Other common pathological features are abnormal muscle fiber differentiation and fiber atrophy and/or hypotrophy. NM is genetically heterogeneous, with disease-causing mutations identified in different genes coding for various components of sarcomeric thin filament.

#### 3.2.2.α-TM

Mutations in the α-TM gene (*TPM1*), mainly expressed in cardiac muscle, have been associated with HCM/DCM (http://cardiogenomics.med.harvard.edu/home). There are currently 13 reported mutations in *TPM1*, of which two are associated with DCM and 11 are associated with HCM (http://www.hgmd.cf.ac.uk/ac/gene.php?gene=TPM1).

#### 3.2.3. β-TM

The *TPM2* isoform of TM encodes for beta-TM, which is mainly expressed in slow, type 1 muscle fibers. Mutations in *TPM2* have recently been identified as an important cause of neuromuscular disorders. These mutations have been associated with various clinical and muscle morphological phenotypes, including congenital NM (E117A), congenital myopathy without rods (Q147P) [75], early onset myopathy with cap structures (E41K and E139del) [76, 77], and congenital joint contractures with distal involvement DA type 1 (R91G) [78], and DA type 2B (R133W) [79]. Different regions of TM are in direct interaction with various thin filament proteins, which may explain why these various mutations lead to different phenotypic expressions.

#### 3.2.4. γ-TM

γ-TM is the product of *TPM3*, which is mainly expressed in slow, type 1 muscle fibers. Mutations in *TPM3* have been associated with both dominant and recessive NM [80–84]. Recently, mutations in *TPM3* have been identified as the common cause of CFTD, a rare form of congenital myopathy with marked type 1 fiber hypotrophy as the main pathological finding on muscle biopsy^85^.

#### 3.2.5. Tn Complex

Mutations in the fast skeletal TnI gene (*TNNI2*) have been associated with DA syndromes. The one missense mutation, Arg174Gln, and the nonsense mutation, Arg156X, have been reported in patients with DA type 2B [78]. In addition, a three-base pair deletion of a glutamate at position 167, has been reported in a three-generation family with DA type 2B [86] and one heterozygous three-base in-frame deletion, Lys176del, has been reported in a three-generation family with DA type 1 [87]. Later a novel three-base in-frame deletion, Lys175del, has been reported in a large Chinese family with DA type 2B [88]. All the reported mutations in the fast skeletal TnI gene associated with DA syndromes have been located in the exon 8 in the carboxy-terminal domain.

Mutations in cardiac TnI (*TNNI3*) have been associated with HCM, DCM and restrictive cardiomyopathy (http://cardiogenomics.med.harvard.edu/home).

So far, no disease has been associated with mutations in the slow skeletal muscle TnI gene (*TNNI1*).

Mutations in the slow skeletal muscle TnT gene (*TNNT1*) are associated with Amish nemaline myopathy (ANM), a form of NM common among the Old Order Amish [89]. Recently, mutations in fast skeletal muscle TnT (*TNNT3*) have been associated with DA type 2B [90]. More than 30 mutations associated with HCM/DCM have been described in the cardiac TnT gene (*TNNT2*) (http://cardiogenomics.med.harvard.edu/home).

So far, no mutation in skeletal muscle TnC has been reported and only a few mutations in cardiac TnC have been associated with HCM/DCM [91, 92].

#### 3.2.6. Nebulin

Mutations in the nebulin gene (*NEB*) are a frequent cause of autosomal recessive NM (OMIM#256030). More than 60 *NEB* mutations have been associated with this form of the rare muscle disorder [93–96]. Recently, homozygous missense mutations in the nebulin gene (*NEB*) have been reported to cause a novel distal myopathy, “Distal nebulin myopathy” [97].

## 4. Conclusions

Despite the significant advances in knowledge of the etiology of skeletal muscle diseases originating in the thick and thin filaments, there is a relative lack of understanding regarding the precise mechanistic links between the primary structural mutations at the skeletal muscle sarcomere level and the defining characteristics of the disorders. Current knowledge will be improved by elucidating novel pathways of disease pathogenesis, required in order to apply biochemical and physiological experimental approaches, anchored in animal models, to clarifying the molecular mechanisms underlying skeletal myopathy pathogenesis.

In order to link genotype to phenotype, a broad range of both *in vivo* and *in vitro* studies focusing on the effects of independent mutations on sarcomeric protein function will be needed; the results may lead to better understanding of the disease process in general and hopefully identify targets for therapeutic intervention.
